# Linking democracy and biodiversity conservation: Empirical evidence and research gaps

**DOI:** 10.1007/s13280-019-01210-0

**Published:** 2019-06-24

**Authors:** Oskar Rydén, Alexander Zizka, Sverker C. Jagers, Staffan I. Lindberg, Alexandre Antonelli

**Affiliations:** 1 Gothenburg Global Biodiversity Centre, Department of Biological and Environmental Sciences, University of Gothenburg, Gothenburg, Sweden; 2 Department of Biological and Environmental Sciences, University of Gothenburg, Box 461, 405 30 Gothenburg, Sweden; 3 Varieties of Democracy Institute, Department of Political Science, University of Gothenburg, Gothenburg, Sweden; 4 German Center for Integrative Biodiversity Research (iDiv), University of Leipzig, Deutscher Platz 5e, 04103 Leipzig, Germany; 5 Department of Political Science, University of Gothenburg, Box 711, 405 30 Gothenburg, Sweden; 6 Centre for Collective Action Research, Department of Political Science, University of Gothenburg, Gothenburg, Sweden; 7 Royal Botanical Gardens Kew, Richmond, Surrey TW9 3AE UK

**Keywords:** Biodiversity proxy, Democratic institutions, Environmental quality, Review, Sustainability

## Abstract

Increasing human pressure threatens plant and animal species with extinction worldwide. National political institutions constitute an important arena for biodiversity conservation. Yet, the relationship between how democratic these national institutions are and a country’s efforts towards and track-record for biodiversity conservation remains poorly understood. In this review, we outline the theoretical links between democracy and biodiversity conservation and review the empirical literature testing them. While more studies reported a positive than a negative relation between democracy and biodiversity conservation (15 vs. 11), the most common result was a mixed relationship (28), often conditioned on economic factors. The use of different proxies to measure biodiversity, including deforestation, protected areas, threatened species, and fishery statistics emerged as a primary obstacle for synthesis. We suggest overcoming this caveat together with a consistent definition of democratic institutions and a standardized statistical framework as research priorities to improve policies against the global biodiversity loss.

**Electronic supplementary material:**

The online version of this article (10.1007/s13280-019-01210-0) contains supplementary material, which is available to authorized users.

## Introduction

Global biological diversity is in crisis. The human need for space and natural resources pushes species extinction rates to all-time highs (e.g. Pimm et al. 2014; Ceballos et al. 2015), well beyond known sustainability levels (e.g. Steffen et al. 2015; Sterner et al. 2019). The conservation of global biodiversity is now identified as an critical challenge for humanity in the twenty-first century among others by the United Nations Sustainable Development Goals (SDG) #14 (“Life Below Water”) and #15 (“Life on Land”) (https://cbd.int/2011-2020/about/sdgs).

While the proximate drivers of this biodiversity loss such as habitat loss, climate change, overexploitation, and invasive species are relatively well-mapped (Brook et al. 2008), less generalizable knowledge exists on the more ultimate causes to those triggers, such as countries’ institutional set-ups (Carpenter et al. 2006; Harmon et al. 2018). Elsewhere, the formal and informal rules shaping the decision-making and the implementation of biodiversity management have been highlighted as paramount (Wells 1998).

This review focuses on the political system and more specifically countries’ democratic institutions at the national level, that is, the formal and informal rules and processes that are shaping how formal political power is accessed and distributed in a given country. For example, free and fair elections is a type of democratic institution that addresses both how political leaders are selected and to whom elected leaders are accountable. While there is a substantial literature on the relevance of political systems for environmental performance (e.g. Dasgupta and De Cian 2018), the specific relationship between democracy and biodiversity conservation is ambiguous and relatively untested. This uncertainty is problematic since regime institutions are mutable and can be targeted for policy actions regarding conservation. Here, we focus on the role of *democracy* rather than the broader concept of *governance*, because, while governance is relevant for conservation (e.g. Barrett et al. 2006; Schulze et al. 2018), the institutions associated with this concept are diffuse and remain conceptually distinct from those normally considered to constitute democracy and differentiate various political regimes (Munck and Verkuilen 2002; Fukuyama 2013).^1^

Political institutions are relevant for biodiversity conservation since the national management of biodiversity can be understood as a case of decision-making in the political system. Thus, variation in the political institutions (i.e. being more or less democratic) that structure the selection of decision-makers, and the processes of decision-making, should be expected to impact the success of biodiversity conservation across countries. Specifically, there are three categories of hypothetical and non-directional arguments why democracy can be related to biodiversity conservation, all of them related to the opportunity structure that actors face (see e.g. Midlarsky 1998; Neumayer 2002; Li and Reuveny 2006 for a more detailed description of the links between democracy, biodiversity conservation, and environmental quality in general).

First, the *political rights*, normally associated with democracy, including the freedom of association, the freedom of expression, and the freedom of press, together allow for a more productive involvement of citizens in politics, both through political parties and civil society organizations and to participate in or lobby decision-making. Political rights also allow for the media and other actors to address biodiversity issues through shaping the public opinion and affecting the policy agenda (Li and Reuveny 2006).

Second, when the faith of political leaders are largely decided by citizens through repeated, free, and fair elections with universal suffrage, the expectation is that this *vertical accountability* electoral mechanism should promote the distribution of environmental public goods (Li and Reuveny 2006), including positive impacts on biodiversity conservation. Additionally, elections as the normal way of selecting leaders tend to reduce short-term uncertainty about political survival (i.e. fear of being removed from office) thus allowing actors to allocate more resources to long-term strategies (Wurster 2013). This can be expected to, for example, promote policies that are better aligned with future needs (i.e. conserving biodiversity for future generations) or allow political parties to compete for support with more or less “green” agendas.

Third, through the *political constraints* that leaders face with increasing liberal democratic institutions, for example the rule of law, judicial constraints, and legislative constraints can foster compliance with legislation and international treaties (Li and Reuveny 2006). Constraining leaders also decreases their possibilities to act opportunistically, which should provide incentives for other actors to cooperate in the management of biodiversity as it introduces stronger mutual expectations of lawful behaviour (Sjöstedt 2013). This may well be expected to have a positive effect on biodiversity.

Note that all of these arguments are more or less procedural and hence non-directional. That is, they may apply both *for* or *against* biodiversity conservation. For instance, *political rights* supply the same opportunity structure to all actors, not just to those who want to protect biodiversity (Midlarsky 1998). *Vertical accountability* can lead to the extraction of natural resources in order to finance specific projects or policies as demanded by voters and consequently supplied by responsive politicians (Desai 1998). It may well also discourage necessary policy action if leaders risk upsetting strategically important actors (Midlarsky 1998) or it can incentivize policies with short-term benefits, instead of having longer time-horizons, due to a need for producing goods that are visible before the next election is scheduled (Lafferty and Meadowcraft 1996, p. 7). *Political constraints* foster policy stability and might thus decrease the possibility for decisive action, which can be negative for biodiversity conservation needs (Wurster 2013). See Table 1 for a simple glossary box of some previously mentioned concepts.

**Table 1:** Glossary box for some concepts

Concept	Can be defined as
Democratic institutions	Used here as a general term to describe the institutions that constitute democracy. Can be understood as the rules and processes (i.e. institutions) that are shaping how formal political power is accessed and distributed in a regime.
Governance	The government’s ability to make and enforce rules, and to deliver services.
Political rights	A set of institutions that distribute the opportunity and freedom for citizens to organize collectively and for the media to operate freely Example: civil society organisations are free to operate, governmental harassments of journalists.
Vertical accountability	A set of institutions that enable citizens to elect political leaders and hold them accountable through electoral means. Example: elected leaders, multiparty electoral competition.
Political constraints	A set of institutions that limits the discretion of elected leaders. Example: autonomous judiciary, a constitution.
Public good	A good that is hard to exclude people from consuming (i.e. non-excludable), and that the availability of this good does not decrease with increasing consumption of it (i.e. non-subtractable). Example: healthy and stable ecosystems.

Following this theoretical line of reasoning, we synthesize the empirical literature on the role of democracy for biodiversity conservation outcomes by

1. *reviewing empirical results* linking biodiversity and democracy at the national level from a comparative perspective;
2. *identify main priorities for future research* on how democracy affects biodiversity based on insights published thus far from environmental and political science.

## Methods

On the 18th and 19th of February, 2019, we conducted a keyword search in the *Web of Science* and *Scopus* databases. We used two keyword strings to generate our sample that was combined using the Boolean AND. For *biodiversity*, we used {biodiversity OR “biodiversity loss” OR “biological conservation” OR “nature conservation” OR “species richness” OR “species extinction risk” OR “species loss” OR “threatened species” OR IUCN OR “ecological sustainability” OR “red list” or “forest loss” OR deforestation OR afforestation OR fisheries OR overfishing OR “environmental commitments” OR “environmental politics” OR “environmental policy” OR “threat status” OR “habitat loss” OR “land use change” OR “protected area”}. For *democracy*, we used {democracy OR autocracy OR democratization OR “democratic governance” OR “democratic institutions” OR authoritarianism OR institutions}.

We limited our search to only include peer-reviewed research articles and reviews in scientific journals written in English and published between 1945 and 2018. The Web of Science and Scopus differ slightly in their search functions. To keep the protocol identical across the databases, we searched for topic in Web of science and for title-abstract-keywords in Scopus. In the former, we restricted the search to the following categories: environmental studies, environmental sciences, ecology, economics, biodiversity conservation, international relations, political science, geography, development studies, forestry, sociology, regional urban planning, water resources, green sustainable science technology, fisheries, public administration, multidisciplinary sciences, social sciences interdisciplinary, and biology. In the latter, we restricted the results to the following categories: environmental sciences, social sciences, agricultural and biological sciences, earth and planetary sciences, economics/econometrics/finance, and multidisciplinary.

**Fig. 1:**
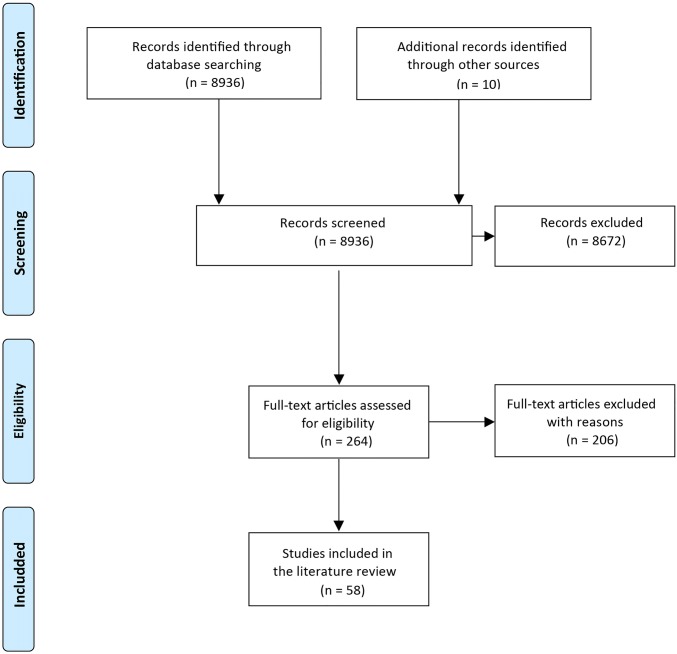
Flow chart of the literature review, modified following Moher et al. 2009. We retained 58 studies out of 8936 records found through Web of Science and Scopus along with the 10 additionally manually added papers

This search returned 8936 items. We scanned the abstracts of these items and retained only those that (1) have a regional scale, i.e. use empirical data from more than two countries and (2) relate any quantitative measure or proxy of biodiversity with any quantitative measure or proxy of democracy in a statistical framework at the national level. We excluded studies using biodiversity as a component of a performance index, as these results survey consistency in environmental performance rather than effects on biodiversity (Scruggs 2009, pp. 4–8). We then removed the duplicates and scanned 264 papers more closely to arrive at a sample of 48 papers. After this procedure, we added 10 additional papers through citation tracking to end up with a sample of 58 papers in total (see supplementary materials).^2^ For the sake of clarity, three individual papers analysed more than one proxy per study. Thus, later on when we sum the sub-totals of how many papers that have worked with each proxy the sum will exceed 58. This happens because we count these three papers once per proxy they use. See Fig. 1 for a summary of the literature review.

We summarize the results in a vote-counting framework by classifying the effect of democracy on biodiversity identified by each study, as positive, negative, mixed, or null. Positive and negative refers to a rather one-sided conclusion based on the empirical analysis, while mixed entails interaction effects, regional disparities, or inconclusive findings. When the overall impact was hard to decide on, we incorporated the conclusions stated in the actual paper into our conclusions. Null represented non-significant findings. In the results section that follows, we highlight the outcomes for the most widely used proxies for biodiversity.

## Results

In the following sections, we present the most commonly used biodiversity proxies in the reviewed studies and their main conclusions. See Table 2 for a summary of the main biodiversity proxies and the supplementary material for all studies that we reviewed. We found a relatively large amount of studies on the democratic determinants of deforestation, represented in 37 papers. The amount of papers analysing the other main proxies was relatively scarce (fisheries = 4, threatened species = 4, and protected areas = 8).

**Table 2:** Biodiversity proxies for linking democracy with biodiversity conservation

Biodiversity proxy	Data availability	Articles	Link to biodiversity	Advantage	Disadvantage
Deforestation	Good	37	Proxy for habitat loss	Global Time-series Data availability	Indirect link to biodiversity Link to biodiversity spatially variable Only indirectly linked to biodiversity
Protected area	Good	8	Proxy for habitat loss	Global Time-series Data availability	Represents commitment rather than state of biodiversity Might be decoupled from biodiversity Biased by socio-economic variables
Red list threat	Medium	4	Per species extinction risk	Global Data availability Direct link to biodiversity loss	Expensive and time intensive Little change through time Temporally limited Taxonomically limited
Marine trophic Index	Good	4	Description of food chain length	Global Time-series Data availability Functional diversity	Indirect link to biodiversity Marine only Only small part of country level biodiversity Link to country borders simplistic
Land-use change	Good	3	Proxy for habitat loss	Global Time-series Data availability	Indirect link to biodiversity
Species abundance	Poor	1	Species number and abundance	Major component of biodiversity Responsive to human pressure Responsive to conservation	Time-lagged response Expensive and time intensive Confounding natural factors No standardized data available
Metabarcoding	Poor	0	Genetic diversity, species number	High taxonomic coverage Relatively cheap Global Time-series	Currently very little data available Method development ongoing

### Deforestation

In our review, nine studies out of 37 associated a higher level of democracy with higher deforestation rates (i.e. a proxied decrease in biodiversity). For instance, a cross-sectional analysis of 75 countries between 1981 and 1990 globally (Midlarsky 1998), in 59 to 74 developing countries between 1990 and 1995 (Marquart-Pyatt 2004; see also Larjavaara 2012), and in a panel of 34 tropical countries between 1972 and 1992 (Dietz and Adger 2003; see also Puzon 2011 for a negative association in a cross-section of Asian and the East Pacific countries). A time-series cross-section analysis of 130 developing countries between 1999 and 2013 found that more autocratic regimes were associated with less economic forest depletion and higher afforestation, although the latter association was less robust than the former (Hermanrud and de Soysa 2017).

In contrast, five studies found a negative association between democracy and deforestation rates (i.e. a positive impact on biodiversity). For instance, a cross-section analysis of the average decadal deforestation rate between 1980 and 2000 indicated that democracy was associated with less deforestation in 134 countries globally (Li and Reuveny 2006). Time-series cross-section analyses further supported this association, also suggesting less deforestation in more democratic contexts in a sample of 59 and 87 (mostly developing) countries between 1972 and 1994, respectively (Nguyen Van and Azomahou 2007; Damette and Delacote 2011).

Furthermore, six studies found no significant association between the level of democracy and deforestation: Exemplified by analyses of the deforestation rate in 59 countries globally between 1972 and 1994 (Damette and Delacote 2012), 74 developing countries between 2001 and 2014 (Restivo et al. 2018), or 55 tropical countries in Latin America, Africa, and Asia between 1980 and 1995 (Bhattarai and Hammig 2004). Another study documented no association between democracy and the yearly change in forest cover for 128 countries between 2001 and 2010. However, the same study found that regime stability (i.e. the number of years since last major change in regime classification) in Sub-Saharan Africa was positively associated with the deforestation rate independent of the initial regime type (Leblois et al. 2017).

Lastly, 17 deforestation studies identified mixed results. For instance, between 1976 and 2003, democracy seemed to be negatively associated with the financial damage from deforestation in Sub-Saharan Africa, Eastern Europe, and the Pacific, but positively so in South Asia, Latin America, and the Caribbean (Arvin and Lew 2011). According to others, between the years 1972 and 1991, democracy was associated with a higher deforestation rate in Latin America and Africa but a lower rate in Asia (Bhattarai and Hammig 2001). A study focusing on Brazil, Indonesia, Malaysia, and the Philippines found that democracy was negatively associated to forest cover change in the former country, but positively so in the three latter countries, during an overall period from 1970 to 1999 (López and Galinato 2005). Different examples of mixed results suggest that democracy might be positively associated with the level of forest coverage but only at higher levels of economic development (Rydning Gaarder and Vadlamannati 2017).

Other conditional associations included international financial characteristics (i.e. foreign direct investment, commodity concentration, and export partner concentration) that were positively associated with the deforestation rate in less democratic contexts (Shandra 2007a), varying associations between democracy and the deforestation rate across different colonial origins (Marchand 2016), a positive association between preventing deforestation and democracy when state capacity was higher (Ehrhardt-Martinez et al. 2002), and interactions between trade openness and regime type (Li and Reuveny 2007). Further, others reported that a positive association between democracy and preventing deforestation is conditional on aggregated psychological factors at the country level (Obydenkova et al. 2016), or that forest governance aid (from Norway) was negatively associated with economic forest depletion when recipient countries already were highly democratic (Hermanrud and de Soysa 2017) and also that a higher amount of environmental civil society organizations per capita was associated with less deforestation in more democratic settings (Shandra et al. 2012). Lastly, there were examples of a non-linear association between democracy and deforestation in both global (Buitenzorgy and Mol 2011; Salahodjaev 2016) and several regional samples (Baliamoune-Lutz 2017; Imai et al. 2018), showing that initial increases in democracy first decrease forest cover, and also that further steps towards democracy are associated with gains in forest cover, suggesting that it is the inconsistent regimes that are deforesting.

### Protected area

In our review, four out of eight articles found a positive link between democracy and protected areas. For instance, the proportion of protected area was positively associated with the level of democracy in a global sample of 100 countries as of 1993 (Midlarsky 1998). Countries that were more democratic had a larger proportion of protected area in 1997, based on multiple measures of democracy in a sample of 145 countries (Neumayer 2002). A recent panel study with data from 1990, 2000, and 2014 and overlapping but somewhat different samples of 115 to 144 countries found a positive association between the level of democracy and the share of national waters under protection (Fouqueray and Papyrakis 2019). A global study of 71 countries between 2000 and 2012 reported that established protected areas were associated with less deforestation in more democratic contexts (Abman 2018).

No study found a negative link between democracy and protected areas, but three studies found mixed results. One study of 137 countries between 1995 and 2012, found a positive association between the level of democracy and terrestrial protected area when economic inequality was lower and, in turn, specifically apply to developing countries (Kashwan 2017). Others showed that political rights and civil liberties interact positively with environmental non-governmental organizations per capita to predict higher proportions of protected areas, based on an analysis of 65 developing countries between 1990 and 2005 (Shandra et al. 2010a). A time-series cross-section analysis of a sample of 130 countries between 1990 and 2005 suggests a positive association between electoral aspects of democracy and the proportion of terrestrial and marine area under protection, but a null association for political constraints (Wurster 2013). One cross-sectional study of 89 countries found a null association between the level of democracy in 1996 and the share of protected areas in 1997 (Nguyen Van 2003).

### Threatened species

Out of four studies, no individual paper documented a clearly positive or negative link between threatened species and democracy. However, three studies found a mixed effect. For instance, a global cross-sectional analysis of 113 countries found that their average level of democracy between 1981 and 2000 was positively associated with the percentage of threatened mammals and birds in 2000, which in the context of this study translated into that democracy was associated with lower fractions of threatened species, although with some differences among taxa (McPherson and Nieswiadomy 2005). Furthermore, a study of 65 developing countries indicated that a higher level of democracy in 1990 was associated with a higher count of threatened mammal species in 2005, but there was a null finding concerning threatened bird species (Shandra et al. 2010a). Another cross-sectional analysis, based on 140 countries in 2010, found that democracy was associated with less threatened mammal, bird, amphibian, reptile, and plant species, but only when economic development was higher (Gren et al. 2016). Finally, one cross-section analysis of threatened mammals in 74 developing nations in the year of 2005 reported a null finding for democratic institutions in 1990 (Shandra et al. 2009).

### Fisheries

In our review, two out of four papers found a positive link between democracy and biodiversity conservation using fisheries data. For instance, one cross-sectional analysis found that the level of democracy was positively associated with the Marine Trophic level Index (MTI, a measure of fish size classes, with low scores indicating overfishing and biodiversity loss) in the exclusive economic zones of coastal Sub-Saharan African countries (Sjöstedt 2013). Another study of the MTI for the same population, stressing the temporal dimension of democracy by reporting the number of years a country had been democratic rather than its current level of democracy, was positively associated to the MTI (Sjöstedt and Jagers 2014).

The remaining two studies found a mixed effect. For instance, a global study on democracy levels and the MTI between 1972 and 2006 suggested a negative association globally, but splitting the samples across income groups showed that the association was negative for a class of poorer countries but positive among more economically developed ones (Povitkina et al. 2015). A recent panel study covering 80 countries with exclusive commercial fishery zones and data from 1986 to 2006 suggested that higher levels of democracy were associated with higher proportions of collapsed fish species, although results were inconsistent across varying model specifications and there was a null finding for overused fish species (Erhardt 2018).

### Other proxies

There were also some alternative but much less used biodiversity proxies. For instance, in one study both democratic and autocratic regime change were associated with the expansion of the agricultural land area, at the cost of natural habitat, albeit with regional differences (Kuusela and Amacher 2016). Another analysis of six South American countries found that agricultural intensification was positively associated with agricultural spatial expansion when the level of democracy was higher (Ceddia et al. 2014). Similarly, when the level of economic development was higher in Brazil, Indonesia, Malaysia, and the Philippines, between 1970 and 1999, democracy was positively associated with paved road expansion (but not crop expansion) into forest-rich regions (López and Galinato 2005).

Two studies analysing countries’ international commitments to conservation suggest a positive association between the level of democracy and compliance with the reporting requirements under the Convention on International Trade in Endangered Species of Wild Fauna and Flora (CITES) in 89 (Carbonell 2016) and in 118 states (Neumayer 2002, using four measures of democracy) in 2000 and 1997. The latter study also showed that the level of democracy in 153 countries was positively associated with states signing the Cartagena Biosafety Protocol in 2000. One analysis constructed a cross-sectional indicator of wetland policy and found a positive association between this and levels of democracy in a sample of 198 countries in 2015 (Peimer et al. 2017). A global analysis of data from 2006 to 2011 showed that more autocratic regimes provided higher proportions of vegetation cover in urban landscapes as compared to more democratic regimes, suggesting better habitat provision (Dobbs et al. 2014).

## Discussion

### Biodiversity indicators

The results of our literature review suggest that biodiversity outcomes in studies related to political systems are mostly based on few indirect proxies. All of them have advantages but also important caveats that are rarely acknowledged.

Deforestation was the most commonly used proxy for biodiversity change. Habitat destruction is a major immediate cause of biodiversity loss (Pimm et al. 2014). In general, the local biodiversity of macro-organisms for an undisturbed habitat will likely be higher than the biodiversity in a similar habitat after strong human disturbance (Barlow et al. 2007), and globally the vegetation of areas with low anthropogenic impact (given sufficient precipitation) are often forests (Van Nes et al. 2014). Hence, deforestation (the loss of forest cover) can serve as a proxy for land-based biodiversity loss on a national scale (Jones et al. 2011). Forest cover is convenient for large-scale analysis because global time-series of forest cover are available going back to at least 1980 (e.g. http://www.fao.org/forestry, http://data.globalforestwatch.org). Despite its prevalence in the empirical literature, we consider forest cover or deforestation rate as a problematic proxy for biodiversity. While highly disturbed habitats might be less diverse than their undisturbed counterparts in general, this is not always the case, and the opposite might even apply (Giam 2017). Additionally, the effect of deforestation on biodiversity might vary across regions and, for instance, depend on the remaining forest cover or the productivity of the area (Dietz and Adger 2003, p. 30).

The amount of area under protection in a country was yet another proxy for biodiversity, in our review most often found to be land-based. The rationale for using the amount of protected area as a proxy for biodiversity is that it can signal a commitment to conservation from governments (Neumayer 2002) and also provide an indication of biodiversity. A major advantage of this proxy is that global time-series data are available, for instance from https://www.protectedplanet.net/. However, there are several caveats that should be mentioned: First, the share of protected area is only indirectly linked to biodiversity as relatively low diversity areas can also be assigned a protected status (Xu et al. 2017). Second, establishing protected areas can be a reaction to an observed decline in biodiversity rather than an indication of sound conservation management (Duit et al. 2009). Third, a large number of legal human activities potentially harmful to biodiversity (e.g. mining, hunting, specific timber extraction, local farming, and hydroelectric power generation) can often take place within protected areas (e.g. Castilho et al. 2017). Fourth, enforcement capacities are limited in many countries (i.e. “paper parks” with low or non-existing de facto protection; cf. Eklund and Cabeza 2017).

The number or fraction of species threatened with extinction following the Red lists of the International Union for the Conservation of Nature, IUCN, (http://www.iucn.org) is another proxy for the level of biodiversity conservation (Table 2). The main advantages of the Red List threat status are its direct link to biodiversity conservation, the relatively good taxonomic and spatial coverage, and the standardized methodology (IUCN Standards and Petitions Subcommittee 2017). On the downside of using threatened species as a proxy for biodiversity, time-series are usually lacking and the number and quality of the status assessment correlate with research effort, which most likely is partially linked to the political and socio-economic factors used in most analyses (Amano and Sutherland 2013). There is thus a risk that systematic bias is present and that the same elements might be included on both sides of the equation, creating circularity.

The biodiversity indicators and proxies presented so far are mostly restricted to terrestrial habitats. An alternative biodiversity proxy for marine systems are fisheries data, often represented by the Marine Trophy Index, MTI. The main advantages of the MTI are its complementary marine perspective, its focus on functional aspects of diversity, the direct response to human pressure (Povitkina et al. 2015), and the excellent data availability over long periods due to its economic relevance (http://www.seaaroundus.org). One caveat of using the MTI as a proxy for biodiversity is that it generally only captures a limited aspect of biodiversity, and marine habitats in territorial waters are usually only a small portion of countries. Furthermore, using MTI at the country level is problematic since marine ecosystems typically interact across regional scales so that policies in one country can affect the measure for other countries (cf. Cash et al. 2006).

Irrespective of the biodiversity proxy, in many cases, the results for linking democracy to biodiversity are mixed. For instance, for deforestation the majority of papers found mixed associations, reporting several conditionality (e.g. economic characteristics, geography, civil society features, state capacity, and non-linear associations), in agreement with a recent meta-analysis on forest governance showing that the inclusion of democracy as a predictor in regression models increases the probability of obtaining inconclusive results (Wehkamp et al. 2018). Most of the other proxies examined also often led to the mixed or null conclusion, except for protected areas for which the associations were mainly positive, although our sample size was small.

One can understand these mixed results through the simple perspective of outcomes and outputs. The former category reflects the state of a given environmental resource while the latter denotes a commitment to that resource (i.e. the difference between establishing a protected area and the fraction of species threatened by extinction). As outputs do not depend as much on geographical or biophysical determinants as outcomes do, they can more easily be related to institutions (Neumayer 2002). As our sample mostly analysed outcomes, this can further explain the mixed findings among these studies and the generally positive conclusions regarding those which explicitly considered outputs.

### Limitations

The link between political regimes and biodiversity conservation is a cross-disciplinary question. It prompts empirical research from both natural and social sciences, including different research approaches, terminology, and methodology. Therefore, we here aimed for a literature review using a vote-count method (i.e. categorizing studies as positive, negative, mixed or null), rather than a formalized meta-analysis, as a first step to combine research and perspectives from both fields. Furthermore, we have focused on processes at the national level because national governments and institutions are the main actors for biodiversity conservation. However, we acknowledge that there might be relevant within-country variation in democracy and biodiversity conservation. Unfortunately, data availability on sub-national democratic institutions limits many comparative analytical enterprises (McMann 2018).

### Priorities for future research

As suggested above, the characteristics of the national level are highly relevant to study for conservation (Harmon et al. 2018), but it is not necessarily sufficient in the face of institutional interplay across different levels. Hence, future research can engage in analysing the interplay between institutions at different levels (Bennett et al. 2017, p. 96) to better understand the inter-scale dynamics, for instance, stakeholder participation at the local level (Young et al. 2012) or the European Union’s efforts through the Natura 2000 project (Blicharska et al. 2016) as means of achieving conservation objectives. The success or failure of these concepts can theoretically be linked to democratic institutions at the national level by acknowledging that they both exist in a hierarchical structure. Integrating the different levels of analysis seems like a promising way to overcome inconsistent empirics and consequently also mixed conclusions.

Here, we suggest three main priorities for further research on the links between democracy and biodiversity that could make conclusions and policy-advice more robust (see Table 3 for a summary). All of these imply a call for more standardized analyses and a more mechanism-based approach towards the causal relationship that theory suggests. These priorities include (1) *biodiversity indicators*, (2) *democratic institutions*, and (3) *model specifications*.

**Table 3:** Priorities for future research on the links between biodiversity and democracy

Priority	Main issue
Biodiversity indicators	(1) Identify or generate standardized indicators that directly capture biodiversity, (2) standardized study-object characteristics (e.g. time period).
Democratic institution	(1) Align democracy measures with conceptual links, (2) use disaggregated democracy measures to better analyse the mechanisms relevant for biodiversity outcomes, (3) study and compare the relative importance of democracy levels, experience with democracy, and regime shifts.
Model specification	Consistently account for (1) confounding factors, (2) conditional relationships, and (3) relevant time-lags between democracy predictors and biodiversity outcomes.

#### Biodiversity indicators

A striking feature of the existing literature is the lack of analyses directly assessing biodiversity at the national scale (cf. Carpenter et al. 2006). Currently, the choice of biodiversity proxy seems mostly driven by data availability, focusing on those presented above with time-series data with global coverage over multiple decades available. However, these proxies are only indirectly linked to biodiversity and have significant weaknesses (see Table 2). Red List assessments over threatened species approximate the state of biodiversity most closely, but their compilation is expensive, time intensive, and they usually change slowly through time. Thus, these are only available for cross-sectional analysis (e.g. McPherson and Nieswiadomy 2005), which is problematic given the likely dynamic component in the structural relationship between democracy and biodiversity (Scruggs 2009).

The generation and use of standardized cross-country time-series indicators that directly quantify terrestrial biodiversity are imperative. Ideally, such biodiversity indicators should directly reflect the state of species populations (i.e. they should indicate abundance), have a standardized global coverage as a time-series, cover a large fraction of the tree of life (many different organism groups, including the “hidden diversity” of e.g. insects, fungi, and microbes), and cover many aspects of biodiversity (e.g. taxonomic, phylogenetic, functional, and ecosystem diversity). Unfortunately, such data do not exist at the larger scale yet, but recent conceptual (e.g. Pereira et al. 2013; Kissling et al. 2018) and data mobilization (e.g. Dornelas et al. 2018) efforts address this issue. Indeed, first global evaluations of the state of biodiversity are undertaken (albeit limited in geographic and taxonomic resolution), such as the Living Planet Index from the World Wildlife Fund (http://livingplanetindex.org/home/index), the State of the World’s Plants and Fungi (https://www.kew.org/science/state-of-the-worlds-plants-and-fungi).

Technological advances might further contribute to ameliorating these issues since novel DNA sequencing techniques allow to measure biodiversity from bulk environmental samples, for instance from lake sediment cores, soil and water samples, and insect traps (e.g. Ritter et al. 2019). Once remaining methodological issues with these technologies are resolved, they hold the potential to revolutionize large-scale biodiversity assessments and provide data to quantify changes in biodiversity through time on a global scale. Until then, at least a standardized reference time interval or baseline to quantify biodiversity loss for biodiversity proxies (e.g. forest cover) could help to reach more robust conclusions across regions and studies.

#### Democratic institutions

Following the theoretical framework laid out in the introduction, there are several pathways between democracy and biodiversity. Since most studies use composite measures that aggregate information on a range of democratic features (e.g. Li and Reuveny 2006), there is little empirical evidence on the relative importance of each of these categories (e.g. Midlarsky 1998; Wehkamp et al. 2018). Hence, we suggest that future work should use more specific democracy indicators to better capture the conceptual links (cf. Sjöstedt and Jagers 2014; Escher and Walter-Rogg 2018).

The majority (59%) of studies in our review use democracy measures provided by the Freedom House (FH) (e.g. Dietz and Adger 2003), the Polity project (e.g. Shandra et al. 2010b), a combination of those (e.g. Erhardt 2018), or the Economist Intelligence Unit (EIU) (e.g. Gren et al. 2016). While these are widely used they do exhibit some shortcomings. For example, FH and EIU aggregate information across the whole political system, including democratic institutions, political culture, government functioning, and even some private market features into single indicators (Munck and Verkuilen 2002; The Economist Intelligence Unit 2017, p. 63ff). This is especially problematic since these factors represent aspects of the political system that are not normally considered to be democratic features. Hence, employing them can lead to conceptual conflation and measurement error consequently generating biased estimates.

The Polity data is focused on democratic features. However, their widely used index, “Polity score” or “Polity2”, omits suffrage from its coding scheme (Munck and Verkuilen 2002). This is concerning because widespread suffrage is one argument as to why democracy is expected to improve biodiversity conservation. Furthermore, low thresholds make the index insensitive to changes in the level of democracy once a first threshold has been reached. For example, the United States of America reached the maximum Polity score in 1815, ignoring all improvements in democratic institutions ever since.

Recent methodological innovations by the Varieties of Democracy Project (http://www.v-dem.net) have made time-series cross-sectional data on highly disaggregated democracy indicators available (Coppedge et al. 2019). The V-Dem data enables researchers to explicitly link the specific democratic institutions suggested by theory at the country level to whatever biodiversity outcome that is of interest for the analyst. For example, this dataset can be used to improve the measurement of democracy by providing better a fit between concepts and constructs or to assess the multidimensionality present in the hypothetical relationship between democratic institutions and biodiversity (cf. Boese 2019 for a recent comparison between measures of democracy).

A second issue with the representation of democracy in empirical studies is that democracy can be related to biodiversity conservation in at least three ways (“modes” hereafter): by its *level* (democratic to autocratic), by the *experience* with (the duration of democratic rule), and by its *stability* (transitioning from one regime type to another) (e.g. Li and Reuveny 2006; Sjöstedt and Jagers 2014; Kuusela and Amacher 2016). In our review, the level of democracy was by far the most commonly used mode, expressed as yearly observations (e.g. Damette and Delacote 2011) or as averages across several years (e.g. McPherson and Nieswiadomy 2005). The experience of democracy was directly analysed in one study, using the age of a democracy (Sjöstedt and Jagers 2014). Stability was examined in three studies through proxies of regime or constitutional change (Deacon 1994; Leblois et al. 2017; Rydning Gaarder and Vadlamannati 2017). The level and experience of democracy are both aligned with the theoretical reasoning but differ in the sense that the former captures the level at a given point in time while the experience taps into institutional legacies and socializing effects, which might be of greater importance (e.g. Sjöstedt and Jagers 2014).

The essence of this segment is that each mode can be related to biodiversity outcomes through distinct mechanisms and thereby have diverging effects. Therefore, inferences across modes should be avoided. In practice, it might be the case that a non-democratic regime becoming democratic is harmful to biodiversity, but that highly democratic countries are better at managing their biological diversity (cf. Walker 1999, p. 263). We suggest to discern the effects of the level, experience, and stability of democracy on biodiversity conservation and to explicitly motivate the importance of each mode in a given case.

#### Model specification

Our review documented a variety of conditional factors and non-linearities regarding the associations between democracy and the biodiversity conservation, ranging from colonial origins, over geographical regions to economic development (e.g. Povitkina et al. 2015; Gren et al. 2016; Marchand 2016). This suggests that unconditional models might be relatively restrictive. Accordingly, future efforts should examine under what circumstances democracy is associated with biodiversity proxies.

A second obstacle to the synthesis of the relationship between democracy and biodiversity was the considerable variation in the sampling of countries. These vary from global and regional samples to tropical, non-core, and developing countries (e.g. Bhattarai and Hammig 2001; Arvin and Lew 2011; Shandra et al. 2011; Ceddia et al. 2014; Sjöstedt and Jagers 2014; Gren et al. 2016). The reasoning behind the different sampling strategies includes, among others, a focus on consistent biogeographic regions (e.g. Dietz and Adger 2003), theoretical relevance (e.g. Shandra et al. 2010a) and statistical considerations (e.g. Ehrhardt-Martinez et al. 2002, p. 233), as well as data availability (e.g. Shandra 2007b). While data selection might be justifiable in some cases, it simultaneously prevents synthesis and generalization.

For example, sampling motivated by economic development can be problematic. Less developed countries also tend to be less democratic (Robinson 2006), restricting the amount of variation in democracy to estimate in the first place. We also hold prior expectations that more developed countries are different from those less developed, so that the relationship between democracy and conservation should vary across them (Povitkina et al. 2015). Lastly, poor areas can overlap geographically with areas relatively rich in biodiversity (Fisher and Christopher 2007), giving them more biodiversity to “lose” comparatively. Given the first point in this paragraph, we can expect less democratic countries to also have higher initial levels of biodiversity that can be lost. These potentially important aspects can be masked if the sampling procedure has discarded the necessary information in order to analyse and communicate them.

It can also be argued that political instability can bias the association between democracy and biodiversity as the latter often coincide with wars and conflict (Hanson et al. 2009) and that less democratic regimes can be associated with an increased risk for political instability (Goldstone et al. 2010). Thus, the relationship between democracy and biodiversity can partially be confounded by political instability events, but it should not fully explain the association (e.g. Reuveny et al. 2010).

A third issue concerns the temporal relationship between democratic institutions and conservation. In our review, time was seldom discussed among the reviewed studies, although it might be highly relevant. The main issues are that the expected time for democracy to affect biodiversity is unclear and that responses of biodiversity to human disturbances can be nearly instantaneous (e.g. hunting or deforestation) or extremely long-term (e.g. competitive exclusion by invasive species, population recovery). We found that democracy was lagged over 1 or up to 15 years, but also in some cases 0 years (e.g. Shandra et al. 2010a; Povitkina et al. 2015; Gren et al. 2016). The averaging strategy minimizes this problem, but introduces other issues such as suitable time periods to average across and a decrease in data points (e.g. Buitenzorgy and Mol 2011).

The omission of relevant dynamics can generate omitted variable bias (De Boef and Keele 2008). According to theory, democracy does not have immediate effects but should instead have a relatively long time-lag (Scruggs 2009, p. 13). However, it is reasonable to expect some difference in the time-lag between, for example, the establishment of protected areas or changes in marine trophic levels when relating these to the democratic institutions (Neumayer 2002, pp. 144–145). While we acknowledge the complexity of incorporating time, it will be a major step towards a better understanding of the democracy–biodiversity relationship.

## Conclusions

Overall, the existing literature on the empirical link between biodiversity conservation and political regimes is ambiguous and important facets for a synthetic understanding are missing. We argue that this is the case partly because of the lack of high-quality data, which forces existing studies to use rough and potentially unsuitable proxies for both biodiversity and democracy, and partly because the mechanisms linking democracy and biodiversity conservation are complex. To address these issues, we suggest as priorities for future research (1) more consistent and relevant indicators for both biodiversity and democracy, (2) a more disaggregated approach to democracy and a mechanistic understanding of how democratic institutions can impact biodiversity conservation, e.g. by using the time-series cross-sectional data on disaggregated democracy indicators provided by the Varieties of Democracy Project, and (3) resolving methodological and theoretical issues relating to sampling, conditionality, and temporal dynamics as crucial priorities for future research.

Both democracy and biodiversity are multidimensional and elusive concepts and therefore complicated subjects for empirical studies. Consequently, this review can only be a starting point for a better understanding. However, the global biodiversity crisis is an issue equally relevant for natural scientists and social scientists, thus solving it is a crucial task for both disciplines and for society at large. We hope that the information and priorities for future research presented here can be a catalyst for cross-disciplinary approaches. Further research on this topic will foster a better understanding on the effect of political regimes on biodiversity conservation and ultimately lead to improved policy approaches.

## Electronic supplementary material

Below is the link to the electronic supplementary material.

Supplementary material 1 (TXT 50 kb)

Supplementary material 2 (PDF 126 kb)

## Footnotes

1 For example, it is a common practice to separate between the institutions that concern *the access* to political power, i.e. democracy, and those associated with *the exercise* of political power, i.e. the bureaucracy and other executive branches (Rothstein 2009).

2 One issue with the sample is the large proportion (19%) of papers (co-)authored by J.M Shandra. While this can indicate productivity it can also overrepresent idiosyncratic assumptions, hence, it must be highlighted.
