# Supplementary material for: Linking democracy and biodiversity conservation: Empirical evidence and research gaps
Source: Ambio. 2019 Jun 24;49(2):419–33. doi: 10.1007/s13280-019-01210-0 (PMC6965076; doi:10.1007/s13280-019-01210-0)
Supplement: Supplementary file 2 — Supplementary material 2 (PDF 126 kb) [file 13280_2019_1210_MOESM2_ESM.pdf]

Electronic supplementary Material for ‘Linking democracy and biodiversity conservation: Empirical evidence and research gaps’

Oskar Rydén, Alexander Zizka, Sverker C. Jagers, Staffan I. Lindberg & Alexandre Antonelli

Appendix 1: Categorization of the reviewed studies

As supplementary data, we include a tab-delimited file ("review_democracy_biodiversity.txt") with each paper as a row, and the topics of interest as columns. In a few cases, some studies are represented on multiple rows as they analyse several biodiversity proxies. There are 58 studies in total with the following 22 variables:

- Reference; Year
- Democracy_measure; Democracy_measure_long
- Averaging; Years_averaged_over
- Democracy_source; Democracy_source_full
- Biodiversity_measure; Biodiversity_measure_full
- Biodiversity_source; Biodiversity_source_full
- Spatial_scale
- Time_start; Time_end; Time_full
- Prediction_effect
- Overall_concluded_impact_of_democracy_on_biodiversity
- Direction_of_the_effect_full
- Stable_link, Comment, Manually_added.

References

Abman, R. 2018. Rule of law and avoided deforestation from protected areas. *Ecological Economics* 146: 282–289. doi:10.1016/j.ecolecon.2017.11.004.

Arvin, M. B., and B. Lew. 2011. Does democracy affect environmental quality in developing countries? *Applied Economics* 43: 1151–1160. doi:10.1080/00036840802600277.

Baliamoune-Lutz, M. 2017. Trade and environmental quality in african countries: Do institutions matter? *Eastern Economic Journal* 43: 155–172. doi:10.1057/s41302-016-0076-8.

Bhattarai, M., and M. Hammig. 2001. Institutions and the environmental Kuznets Curve for

deforestation: A crosscountry analysis for Latin America, Africa and Asia. *World Development* 29: 995–1010. doi:10.1016/S0305-750X(01)00019-5.

Bhattarai, M., and M. Hammig. 2004. Governance, economic policy, and the environmental Kuznets curve for natural tropical forests. *Environment and Development Economics* 9: 367–382. doi:10.1017/S1355770X03001293.

Carbonell, J. R. 2016. Military spending, liberal institutions and state compliance with international environmental agreements. *International Environmental Agreements: Politics, Law and Economics* 16: 691–719. doi:10.1007/s10784-015-9290-9.

Ceddia, M. G., N. O. Bardsley, S. Gomez-y-Paloma, and S. Sedlacek. 2014. Governance, agricultural intensification, and land sparing in tropical South America. *Proceedings of the National academy of Sciences of the United States of America* 111: 7242–7247. doi:10.1073/pnas.1317967111.

Damette, O., and P. Delacote. 2011. Unsustainable timber harvesting, deforestation and the role of certification. *Ecological Economics* 70: 1211–1219. doi:10.1016/j.ecolecon.2011.01.025.

Damette, O., and P. Delacote. 2012. On the economic factors of deforestation: What can we learn from quantile analysis? *Economic Modelling* 29: 2427–2434. doi:10.1016/j.econmod.2012.06.015.

Deacon, R. T. 1994. Deforestation and the rule of law in a cross-section of countries. *Land Economics* 70: 414–430.

Didia, D. 1997. Democracy, political instability and tropical deforestation. *Global Environmental Change* 7: 63–76. doi:10.1016/S0959-3780(96)00024-6.

Dietz, S., and W. N. Adger. 2003. Economic growth, biodiversity loss and conservation effort. *Journal of Environmental Management* 68: 23–35. doi:10.1016/S0301-4797(02)00231-1.

Dobbs, C., C. R. Nitschke, and D. Kendal. 2014. Global drivers and tradeoffs of three urban vegetation ecosystem services. *PLoS ONE* 9: e113000. doi:10.1371/journal.pone.0113000.

Ehrhardt-Martinez, K., E. M. Crenshaw, and J. C. Jenkins. 2002. Deforestation and the environmental Kuznets curve: A cross national investigation of intervening mechanisms. *Social Science Quarterly* 83: 226–243. doi:10.1111/1540-6237.00080.

Erhardt, T. 2018. Does International Trade Cause Overfishing? *Journal of the Association of Environmental and Resource Economists* 5: 695–711. doi:10.1086/698362.

Fouqueray, M., and E. Papyrakis. 2019. An empirical analysis of the cross-national determinants of marine protected areas. *Marine Policy* 99: 87–93. doi:10.1016/j.marpol.2018.10.017.

Gren, I. M., M. Campos, and L. Gustafsson. 2016. Economic development, institutions, and biodiversity loss at the global scale. *Regional Environmental Change* 16: 445–457. doi:10.1007/s10113-015-0754-9.

Hermanrud, K., and I. de Soysa. 2017. Lazy thinking, lazy giving? Examining the effects of development aid on forests in developing countries. *International Area Studies Review* 20: 19–41. doi:10.1177/2233865916682430.

Imai, N., T. Furukawa, R. Tsujino, S. Kitamura, and T. Yumoto. 2018. Factors affecting forest area change in southeast Asia during 1980–2010. *PLoS ONE* 13: 1–14. doi:10.1371/journal.pone.0197391.

Kashwan, P. 2017. Inequality, democracy, and the environment: A cross-national analysis. *Ecological Economics* 131: 139–151. doi:10.1016/j.ecolecon.2016.08.018.

Kuusela, O. P., and G. S. Amacher. 2016. Changing political regimes and tropical deforestation. *Environmental & Resource Economics* 64: 445–463. doi:10.1007/s10640-015-9880-6.

Larjavaara, M. 2012. Democratic less-developed countries cause global deforestation. *International Forestry Review* 14: 299–313. doi:10.1505/146554812802646666.

Leblois, A., O. Damette, and J. Wolfersberger. 2017. What has driven deforestation in developing countries since the 2000s? Evidence from new remote-sensing data. *World Development* 92: 82–102. doi:10.1016/j.worlddev.2016.11.012.

Li, Q., and R. Reuveny. 2006. Democracy and environmental degradation. *International Studies Quarterly* 50: 935–956. doi:10.1111/j.1468-2478.2006.00432.x.

Li, Q., and R. Reuveny. 2007. The effects of liberalism on the terrestrial environment. *Conflict Management and Peace Science* 24: 219–238. doi:10.1080/07388940701468492.

López, R., and G. I. Galinato. 2005. Deforestation and forest-induced carbon dioxide emissions in tropical countries: How do governance and trade openness affect the forest–income relationship? *Journal of Environment and Development* 14: 73–100. doi:10.1177/1070496504273878.

Marchand, S. 2016. The colonial origins of deforestation: An institutional analysis. *Environment and Development Economics* 21: 318–349. doi:10.1017/S1355770X1500025X.

Marquart-Pyatt, S. 2004. A cross-national investigation of deforestation, debt, state fiscal capacity, and the Environmental Kuznets Curve. *International Journal of Sociology* 34: 33–51. doi:10.1080/00207659.2004.11043128.

Mather, A. S., and C. L. Needle. 1999. Development, democracy and forest trends. *Global Environmental Change* 9: 105–118. doi:10.1016/S0959-3780(98)00035-1.

McPherson, M. A., and M. L. Nieswiadomy. 2005. Environmental Kuznets Curve: Threatened species and spatial effects. *Ecological Economics* 55: 395–407. doi:10.1016/j.ecolecon.2004.12.004.

Midlarsky, M. 1998. Democracy and the environment: An empirical assessment. *Journal of Peace Research* 35: 341–361. doi:http://www.jstor.org/stable/424940.

Neumayer, E. 2002. Do democracies exhibit stronger international environmental commitment? A cross-country analysis. *Journal of Peace Research* 39: 139–164. doi:10.1177/0022343302039002001.

Nguyen Van, P. 2003. A semiparametric analysis of determinants of a protected area. *Applied Economics Letters* 10: 661–665. doi:10.1080/1350485032000129098.

Nguyen Van, P., and T. Azomahou. 2007. Nonlinearities and heterogeneity in environmental

quality: An empirical analysis of deforestation. *Journal of Development Economics* 84: 291–309. doi:10.1016/j.jdeveco.2005.10.004.

Obydenkova, A., Z. Nazarov, and R. Salahodjaev. 2016. The process of deforestation in weak democracies and the role of intelligence. *Environmental Research* 148: 484–490. doi:10.1016/j.envres.2016.03.039.

Peimer, A. W., A. E. Krzywicka, D. B. Cohen, K. Van den Bosch, V. L. Buxton, N. A. Stevenson, and J. W. Matthews. 2017. National-level wetland policy specificity and goals vary according to political and economic indicators. *Environmental Management* 59: 141–153. doi:10.1007/s00267-016-0766-3.

Povitkina, M., S. C. Jagers, M. Sjöstedt, and A. Sundström. 2015. Democracy, development and the marine environment - A global time-series investigation. *Ocean and Coastal Management* 105: 25–34. doi:10.1016/j.ocecoaman.2014.12.023.

Puzon, K. 2011. Deforestation, economic prosperity, and political institutions in East Asia and the Pacific. *International Journal of Green Economics* 5: 248. doi:10.1504/ijge.2011.044236.

Restivo, M., J. M. Shandra, and J. M. Sommer. 2018. The United States Agency for International Development and forest loss: A cross-national analysis of environmental aid. *The Social Science Journal* 55: 171–181. doi:10.1016/j.soscij.2017.09.001.

Reuveny, R., A. S. Mihalache-O'Keef, and Q. Li. 2010. The effect of warfare on the environment. *Journal of Peace Research* 47: 749–761. doi:10.1177/0022343310382069.

Rydning Gaarder, A., and K. C. Vadlamannati. 2017. Does democracy guarantee (de)forestation? An empirical analysis. *International Area Studies Review* 20: 97–121. doi:10.1177/2233865916688846.

Salahodjaev, R. 2016. Intelligence and deforestation: International data. *Forest Policy and Economics* 63: 20–27. doi:10.1016/j.forpol.2015.12.003.

Shandra, J. M. 2007a. Economic dependency, repression, and deforestation: A quan-

titative, cross-national analysis. *Sociological Inquiry* 77: 543–571. doi:10.1111/j.1475-682X.2007.00209.x.

Shandra, J. M. 2007b. International nongovernmental organizations and deforestation: Good, bad, or irrelevant? *Social Science Quarterly* 88: 665–689. doi:10.1111/j.1540-6237.2007.00477.x.

Shandra, J. M. 2007c. The world polity and deforestation: A quantitative, cross-national analysis. *International Journal of Comparative Sociology* 48: 5–27. doi:10.1177/0020715207072157.

Shandra, J. M., C. L. Shandra, and B. London. 2008. Women, non-governmental organizations, and deforestation: A cross-national study. *Population and Environment* 30: 48–72. doi:10.1007/s11111-008-0073-x.

Shandra, J. M., C. Leckband, L. A. McKinney, and B. London. 2009. Ecologically unequal exchange, world polity, and biodiversity loss: A cross-national analysis of threatened mammals. *International Journal of Comparative Sociology* 50: 285–310. doi:10.1177/0020715209105143.

Shandra, J. M., A. L. McKinney, C. Leckband, and B. London. 2010. Debt, Structural adjustment, and Biodiversity loss: a cross-national analysis of threatend mammals and birds. *Human Ecology Review* 17: 18–33. doi:https://www.jstor.org/stable/24707512.

Shandra, J. M., T. K. Rudel, M. Restivo, and B. London. 2010. Nongovernmental organizations and protected land area. *International Journal of Sociology* 40: 85–99. doi:10.2753/IJS0020-7659400205.

Shandra, J. M., M. Restivo, E. Shircliff, and B. London. 2011. Do commercial debt-for-nature swaps matter for forests? A cross-national test of world polity theory. *Sociological Forum* 26: 381–410. doi:doi.org/10.1111/j.1573-7861.2011.01245.x.

Shandra, J. M., E. Shircliff, and B. London. 2011. World Bank lending and deforestation: A cross-national analysis. *International Sociology* 26: 292–314. doi:10.1177/0268580910392260.

Shandra, J. M., L. E. Esparza, and B. London. 2012. Nongovernmental organizations,

democracy, and deforestation: A cross-national analysis. *Society and Natural Resources* 25: 251–269. doi:10.1080/08941920.2011.573841.

Sjöstedt, M. 2013. Horizontal and vertical resource dilemmas in natural resource management: The case of African fisheries. *Fish and Fisheries* 14: 616–624. doi:10.1111/j.1467-2979.2012.00481.x.

Sjöstedt, M., and S. C. Jagers. 2014. Democracy and the environment revisited: The case of African fisheries. *Marine Policy* 43: 143–148. doi:10.1016/j.marpol.2013.05.007.

Wehkamp, J., N. Koch, S. Lübbers, and S. Fuss. 2018. Governance and deforestation—A meta-analysis in economics. *Ecological Economics* 144: 214–227. doi:10.1016/j.ecolecon.2017.07.030.

Wurster, S. 2013. Comparing ecological sustainability in autocracies and democracies. *Contemporary Politics* 19: 76–93. doi:10.1080/13569775.2013.773204.
